# 3-Ethyl 5-methyl 2-hydr­oxy-6-methyl-4-(4-nitro­phen­yl)-2-trifluoro­methyl-1,2,3,4-tetra­hydro­pyridine-3,5-dicarboxyl­ate

**DOI:** 10.1107/S1600536808024835

**Published:** 2008-08-09

**Authors:** Chen-Xia Yu, Pei-Li Qian, Jia-Jing Ping, Chang-Sheng Yao

**Affiliations:** a School of Chemistry and Chemical Engineering, Xuzhou Normal University, Xuzhou 221116, People’s Republic of China; b Key Laboratory of Biotechnology for Medicinal Plants, Xuzhou Normal University, Xuzhou 221116, People’s Republic of China; c Kewen College, Xuzhou Normal University, Xuzhou 221116, People’s Republic of China

## Abstract

In the title compound, C_18_H_19_F_3_N_2_O_7_, the tetrahydropyridine ring adopts a half-chair conformation. The nitro group is disordered over two sites with occupancies of 0.780 (15) and 0.220 (15). An intra­molecular N—H⋯F hydrogen bond is observed in the mol­ecular structure. The mol­ecules are linked into a two-dimensional network parallel to (100) by O—H⋯O, N—H⋯O and C—H⋯O hydrogen bonds.

## Related literature

For related literature, see: Achiwa & Kato (1999 ▶); Dubur *et al.* (1989 ▶); Hermann *et al.* (2003 ▶); Ulrich (2004 ▶).
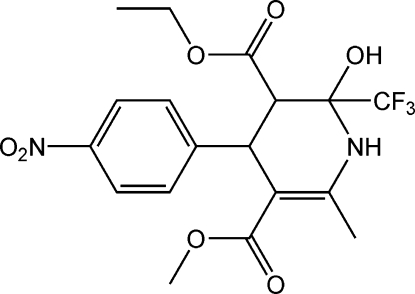


## Experimental

### 

#### Crystal data

C_18_H_19_F_3_N_2_O_7_
*M* *_r_* = 432.35
Monoclinic, 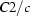

*a* = 28.678 (6) Å
*b* = 9.6678 (19) Å
*c* = 14.120 (3) Å
β = 95.72 (3)°
*V* = 3895.1 (13) Å^3^
*Z* = 8
Mo *K*α radiation
μ = 0.13 mm^−1^
*T* = 293 (2) K
0.16 × 0.16 × 0.04 mm

#### Data collection

Rigaku Saturn diffractometer
Absorption correction: multi-scan (*CrystalClear*; Rigaku/MSC, 2002 ▶) *T*_min_ = 0.979, *T*_max_ = 0.995
11673 measured reflections
3438 independent reflections
2572 reflections with *I* > 2σ(*I*)
*R*_int_ = 0.043

#### Refinement

*R*[*F* ^2^ > 2σ(*F* ^2^)] = 0.052
*wR*(*F* ^2^) = 0.150
*S* = 1.06
3438 reflections
284 parameters
14 restraints
H-atom parameters constrained
Δρ_max_ = 0.22 e Å^−3^
Δρ_min_ = −0.20 e Å^−3^

### 

Data collection: *CrystalClear* (Rigaku/MSC, 2002 ▶); cell refinement: *CrystalClear*; data reduction: *CrystalClear*; program(s) used to solve structure: *SHELXS97* (Sheldrick, 2008 ▶); program(s) used to refine structure: *SHELXL97* (Sheldrick, 2008 ▶); molecular graphics: *SHELXTL* (Sheldrick, 2008 ▶); software used to prepare material for publication: *SHELXTL*.

## Supplementary Material

Crystal structure: contains datablocks I, global. DOI: 10.1107/S1600536808024835/ci2642sup1.cif

Structure factors: contains datablocks I. DOI: 10.1107/S1600536808024835/ci2642Isup2.hkl

Additional supplementary materials:  crystallographic information; 3D view; checkCIF report

## Figures and Tables

**Table 1:** Hydrogen-bond geometry (Å, °)

*D*—H⋯*A*	*D*—H	H⋯*A*	*D*⋯*A*	*D*—H⋯*A*
O7—H7⋯O5^i^	0.82	1.98	2.783 (3)	166
N2—H2⋯O3^ii^	0.86	2.20	3.030 (3)	163
N2—H2⋯F2	0.86	2.42	2.735 (2)	102
C5—H5⋯O2^iii^	0.93	2.51	3.432 (4)	171

Symmetry codes: (i) 
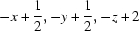
; (ii) 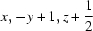; (iii) 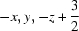.

**Fig. 1.**
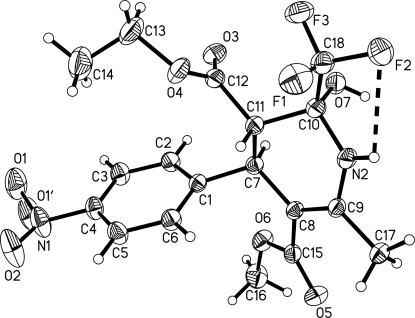
The molecular structure of the title compound, showing 30% probability displacement ellipsoids and the atom-numbering scheme. Both disorder components are shown. The dashed line indicates a hydrogen bond.

**Fig. 2.**
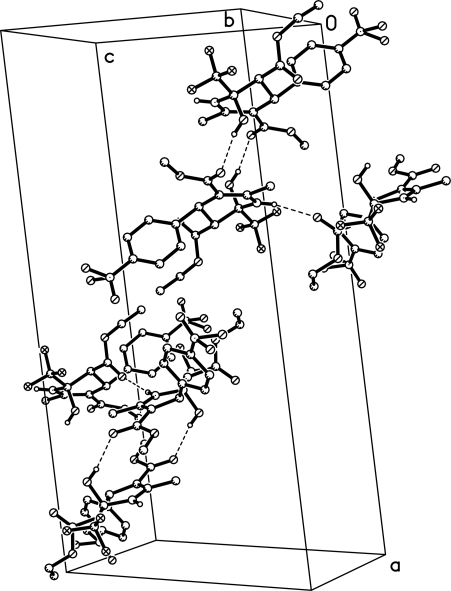
The packing diagram of the title compound. Intermolecular hydrogen bonds are shown as dashed lines and H atoms not involved in hydrogen bonding have been omitted for clarity.

## supplementary crystallographic information

### Comment

1,4-Dihydropyridines are well known compounds as a consequence of their pharmacological profile as the most important calcium channel modulators (Achiwa & Kato, 1999). 4-Aryl-2,6-dimethyl-1,4-dihydropyridine -3,5-dicarboxylate derivatives are widely used for the treatment of cardiovascular diseases such as hypertension, angina pectoris and infarction (Dubur *et al.*, 1989). In addition, compounds that contain fluorine have special bioactivity, for example, flumioxazin is a widely used herbicide (Hermann *et al.*, 2003; Ulrich,2004). This led us to pay much attention to the synthesis and bioactivity of these fluoro-compounds. During the synthesis of trifluoromethylated 1,4-dihydropyridine derivatives, an intermediate, the title compound, was isolated. We report here the crystal structure of the title compound.

In the title molecule (Fig.1), the pyridine ring adopts a half-chair conformation, with atoms C10 and C11 deviating from the N2/C7/C8/C9 plane by 0.261 (4) Å and -0.544 (4)Å, respectively. The dihedral angle between the N2/C7/C8/C9 and C1-C6 planes is 72.30 (9)°. The N atom of the nitro group adopts a planar configuration in the major disorder component, while pyramidal configuration in the minor disorder component. An intramolecular N2—H2···F2 hydrogen bond is observed in the molecular structure.

The crystal packing is stabilized by O—H···O, N—H···O and C—H···O intermolecular hydrogen bonds (Table 1) which link the molecules into a two-dimensional network parallel to the (1 0 0) (Fig. 2).

### Experimental

The title compound was synthesized by the reaction of 4-nitrobenzaldehyde (1 mmol), methyl 3-amino-but-2-enoate (1 mmol) and ethyl 4,4,4-trifluoro-3-oxobutanoate (1 mmol) catalyzed by triethylbenzylaminium chloride (0.02 g) in water (10 ml) at 363 K. After cooling, the reaction mixture was washed with water and recrystallized from ethanol, to obtain single crystals suitable for X-ray diffraction.

### Refinement

All H atoms were placed in calculated positions (C-H = 0.93–0.98 Å, O-H = 0.82 Å and N-H = 0.86 Å) and included in the final cycles of refinement using a riding model, with *U*_iso_(H) = 1.2*U*_eq_(parent atom). Atom O1 is disordered over two positions with site-occupancy factors of 0.220 (15) and 0.780 (15), respectively. The *U^ij^* components of disordered atoms were restrained to an approximate isotropic behaviour. The N1—O1 and N1—O1' bond lengths were restrained to 1.22 (2) Å.

### Crystal data

**Table:**

C_18_H_19_F_3_N_2_O_7_	*F*_000_ = 1792
*M**_r_* = 432.35	*D*_x_ = 1.475 Mg m^−^^3^
Monoclinic, *C*2/*c*	Mo *K*α radiation λ = 0.71073 Å
Hall symbol: -C 2yc	Cell parameters from 3562 reflections
*a* = 28.678 (6) Å	θ = 2.2–25.0º
*b* = 9.6678 (19) Å	µ = 0.13 mm^−^^1^
*c* = 14.120 (3) Å	*T* = 293 (2) K
β = 95.72 (3)º	Prism, colourless
*V* = 3895.1 (13) Å^3^	0.16 × 0.16 × 0.04 mm
*Z* = 8	

### Data collection

**Table:**

Rigaku Saturn diffractometer	3438 independent reflections
Radiation source: rotating anode	2572 reflections with *I* > 2σ(*I*)
Monochromator: confocal	*R*_int_ = 0.043
Detector resolution: 7.31 pixels mm^-1^	θ_max_ = 25.0º
*T* = 293(2) K	θ_min_ = 2.2º
ω scans	*h* = −33→34
Absorption correction: multi-scan(CrystalClear; Rigaku/MSC, 2002)	*k* = −11→11
*T*_min_ = 0.979, *T*_max_ = 0.995	*l* = −16→12
11673 measured reflections	

### Refinement

**Table:**

Refinement on *F*^2^	Secondary atom site location: difference Fourier map
Least-squares matrix: full	Hydrogen site location: inferred from neighbouring sites
*R*[*F*^2^ > 2σ(*F*^2^)] = 0.052	H-atom parameters constrained
*wR*(*F*^2^) = 0.150	*w* = 1/[σ^2^(*F*_o_^2^) + (0.0873*P*)^2^] where *P* = (*F*_o_^2^ + 2*F*_c_^2^)/3
*S* = 1.06	(Δ/σ)_max_ = 0.001
3438 reflections	Δρ_max_ = 0.22 e Å^−^^3^
284 parameters	Δρ_min_ = −0.20 e Å^−^^3^
14 restraints	Extinction correction: none
Primary atom site location: structure-invariant direct methods	

### Special details

**Table:**

Geometry. All e.s.d.'s (except the e.s.d. in the dihedral angle between two l.s. planes) are estimated using the full covariance matrix. The cell e.s.d.'s are taken into account individually in the estimation of e.s.d.'s in distances, angles and torsion angles; correlations between e.s.d.'s in cell parameters are only used when they are defined by crystal symmetry. An approximate (isotropic) treatment of cell e.s.d.'s is used for estimating e.s.d.'s involving l.s. planes.
Refinement. Refinement of *F*^2^ against ALL reflections. The weighted *R*-factor *wR* and goodness of fit *S* are based on *F*^2^, conventional *R*-factors *R* are based on *F*, with *F* set to zero for negative *F*^2^. The threshold expression of *F*^2^ > σ(*F*^2^) is used only for calculating *R*-factors(gt) *etc*. and is not relevant to the choice of reflections for refinement. *R*-factors based on *F*^2^ are statistically about twice as large as those based on *F*, and *R*- factors based on ALL data will be even larger.

### Fractional atomic coordinates and isotropic or equivalent isotropic displacement parameters (Å^2^)

**Table:**

	*x*	*y*	*z*	*U*_iso_*/*U*_eq_	Occ. (<1)
F1	0.08689 (6)	0.58977 (17)	0.93658 (13)	0.0775 (5)	
F2	0.15032 (7)	0.70065 (15)	0.97856 (12)	0.0800 (6)	
F3	0.12464 (6)	0.68534 (15)	0.83137 (11)	0.0725 (5)	
O1	0.0452 (7)	−0.100 (2)	0.5112 (8)	0.086 (6)	0.220 (15)
O1'	0.06713 (19)	−0.1837 (7)	0.5322 (3)	0.090 (2)	0.780 (15)
O2	0.01478 (8)	−0.1793 (3)	0.63009 (19)	0.0972 (9)	
O3	0.13847 (6)	0.44788 (18)	0.67834 (12)	0.0545 (5)	
O4	0.06795 (6)	0.44145 (19)	0.73469 (12)	0.0594 (5)	
O5	0.21673 (6)	0.00503 (18)	1.01751 (13)	0.0546 (5)	
O6	0.21490 (6)	0.01870 (17)	0.85947 (12)	0.0512 (5)	
O7	0.19972 (6)	0.52220 (18)	0.86560 (12)	0.0512 (5)	
H7	0.2218	0.5189	0.9071	0.077*	
N1	0.05038 (9)	−0.1349 (3)	0.6024 (2)	0.0738 (8)	
N2	0.16656 (7)	0.42268 (18)	0.99703 (13)	0.0432 (5)	
H2	0.1630	0.4725	1.0463	0.052*	
C1	0.13495 (7)	0.1409 (2)	0.77500 (16)	0.0353 (5)	
C2	0.14347 (8)	0.1173 (2)	0.68116 (16)	0.0424 (6)	
H2A	0.1683	0.1621	0.6565	0.051*	
C3	0.11549 (9)	0.0281 (2)	0.62409 (18)	0.0485 (6)	
H3	0.1210	0.0132	0.5611	0.058*	
C4	0.07957 (8)	−0.0378 (2)	0.66212 (18)	0.0469 (6)	
C5	0.07026 (9)	−0.0190 (3)	0.75489 (19)	0.0502 (6)	
H5	0.0458	−0.0659	0.7793	0.060*	
C6	0.09829 (8)	0.0715 (2)	0.81067 (17)	0.0446 (6)	
H6	0.0924	0.0860	0.8735	0.053*	
C7	0.16340 (7)	0.2485 (2)	0.83367 (15)	0.0365 (5)	
H7A	0.1902	0.2747	0.7996	0.044*	
C8	0.18168 (7)	0.2018 (2)	0.93315 (15)	0.0354 (5)	
C9	0.18004 (7)	0.2873 (2)	1.00940 (15)	0.0368 (5)	
C10	0.15830 (8)	0.4834 (2)	0.90448 (17)	0.0427 (6)	
C11	0.13175 (8)	0.3763 (2)	0.83985 (16)	0.0373 (5)	
H11	0.1044	0.3473	0.8711	0.045*	
C12	0.11414 (9)	0.4275 (2)	0.74130 (17)	0.0418 (6)	
C13	0.04231 (12)	0.4735 (4)	0.6429 (2)	0.0755 (9)	
H13A	0.0637	0.5090	0.5997	0.091*	
H13B	0.0189	0.5439	0.6509	0.091*	
C14	0.01957 (13)	0.3484 (4)	0.6032 (2)	0.0918 (11)	
H14A	0.0430	0.2830	0.5884	0.138*	
H14B	−0.0001	0.3712	0.5462	0.138*	
H14C	0.0008	0.3086	0.6488	0.138*	
C15	0.20522 (8)	0.0677 (2)	0.94376 (17)	0.0396 (5)	
C16	0.23732 (10)	−0.1148 (3)	0.8569 (2)	0.0651 (8)	
H16A	0.2607	−0.1231	0.9101	0.098*	
H16B	0.2518	−0.1237	0.7987	0.098*	
H16C	0.2143	−0.1864	0.8601	0.098*	
C17	0.19319 (8)	0.2520 (2)	1.11186 (15)	0.0460 (6)	
H17A	0.1907	0.3331	1.1502	0.069*	
H17B	0.2249	0.2184	1.1198	0.069*	
H17C	0.1725	0.1818	1.1313	0.069*	
C18	0.12955 (10)	0.6156 (2)	0.91287 (19)	0.0543 (7)	

### Atomic displacement parameters (Å^2^)

**Table:**

	*U*^11^	*U*^22^	*U*^33^	*U*^12^	*U*^13^	*U*^23^
F1	0.0761 (12)	0.0770 (11)	0.0819 (13)	0.0222 (9)	0.0199 (10)	−0.0030 (9)
F2	0.1242 (15)	0.0509 (9)	0.0612 (11)	0.0076 (9)	−0.0094 (10)	−0.0176 (8)
F3	0.1081 (13)	0.0524 (9)	0.0544 (10)	0.0161 (8)	−0.0046 (9)	0.0094 (7)
O1	0.085 (8)	0.118 (10)	0.055 (7)	−0.032 (7)	0.008 (5)	−0.026 (6)
O1'	0.093 (3)	0.111 (4)	0.069 (2)	−0.038 (3)	0.023 (2)	−0.052 (2)
O2	0.0728 (14)	0.1166 (18)	0.1066 (19)	−0.0437 (14)	0.0303 (14)	−0.0568 (16)
O3	0.0674 (11)	0.0622 (11)	0.0344 (10)	−0.0019 (8)	0.0074 (9)	0.0101 (8)
O4	0.0528 (11)	0.0799 (12)	0.0425 (11)	0.0022 (9)	−0.0094 (9)	0.0098 (9)
O5	0.0621 (11)	0.0555 (10)	0.0458 (11)	0.0078 (8)	0.0028 (9)	0.0114 (9)
O6	0.0595 (11)	0.0521 (10)	0.0420 (10)	0.0109 (8)	0.0042 (9)	−0.0055 (8)
O7	0.0536 (10)	0.0592 (10)	0.0403 (10)	−0.0162 (9)	0.0018 (8)	0.0045 (8)
N1	0.0681 (16)	0.0885 (18)	0.0668 (18)	−0.0311 (14)	0.0167 (14)	−0.0349 (15)
N2	0.0620 (13)	0.0418 (10)	0.0254 (10)	0.0000 (9)	0.0027 (9)	−0.0041 (8)
C1	0.0372 (12)	0.0379 (11)	0.0307 (12)	0.0012 (9)	0.0025 (10)	−0.0011 (9)
C2	0.0468 (13)	0.0470 (13)	0.0348 (13)	−0.0064 (11)	0.0109 (11)	−0.0042 (11)
C3	0.0543 (15)	0.0555 (14)	0.0368 (14)	−0.0044 (12)	0.0099 (12)	−0.0133 (11)
C4	0.0476 (14)	0.0504 (13)	0.0425 (14)	−0.0070 (11)	0.0032 (12)	−0.0148 (11)
C5	0.0478 (14)	0.0553 (14)	0.0487 (15)	−0.0135 (12)	0.0103 (12)	−0.0045 (12)
C6	0.0506 (14)	0.0524 (13)	0.0318 (13)	−0.0075 (11)	0.0097 (11)	−0.0045 (11)
C7	0.0392 (12)	0.0414 (11)	0.0291 (11)	−0.0038 (9)	0.0047 (10)	0.0004 (10)
C8	0.0356 (11)	0.0421 (11)	0.0286 (12)	−0.0036 (9)	0.0028 (9)	0.0022 (9)
C9	0.0360 (11)	0.0447 (12)	0.0294 (12)	−0.0058 (10)	0.0008 (9)	0.0023 (10)
C10	0.0536 (14)	0.0420 (12)	0.0320 (13)	−0.0026 (10)	0.0022 (11)	0.0012 (10)
C11	0.0420 (12)	0.0408 (11)	0.0291 (12)	−0.0032 (10)	0.0030 (10)	0.0001 (9)
C12	0.0521 (14)	0.0390 (12)	0.0338 (13)	−0.0027 (10)	0.0016 (12)	0.0007 (10)
C13	0.076 (2)	0.095 (2)	0.0499 (18)	0.0053 (18)	−0.0195 (16)	0.0128 (17)
C14	0.091 (2)	0.114 (3)	0.065 (2)	−0.019 (2)	−0.0149 (19)	−0.008 (2)
C15	0.0373 (12)	0.0449 (12)	0.0363 (13)	−0.0063 (10)	0.0015 (10)	−0.0001 (11)
C16	0.0681 (18)	0.0536 (15)	0.072 (2)	0.0125 (13)	0.0009 (16)	−0.0161 (14)
C17	0.0544 (14)	0.0520 (13)	0.0309 (13)	−0.0051 (11)	0.0000 (11)	0.0020 (11)
C18	0.0761 (19)	0.0455 (14)	0.0403 (15)	0.0047 (13)	0.0008 (14)	−0.0029 (12)

### Geometric parameters (Å, °)

**Table:**

F1—C18	1.324 (3)	C5—C6	1.380 (3)
F2—C18	1.335 (3)	C5—H5	0.93
F3—C18	1.329 (3)	C6—H6	0.93
O1—N1	1.325 (13)	C7—C8	1.518 (3)
O1'—N1	1.237 (4)	C7—C11	1.541 (3)
O2—N1	1.208 (3)	C7—H7A	0.98
O3—C12	1.200 (3)	C8—C9	1.362 (3)
O4—C12	1.326 (3)	C8—C15	1.462 (3)
O4—C13	1.458 (3)	C9—C17	1.498 (3)
O5—C15	1.221 (3)	C10—C18	1.532 (3)
O6—C15	1.336 (3)	C10—C11	1.532 (3)
O6—C16	1.444 (3)	C11—C12	1.515 (3)
O7—C10	1.408 (3)	C11—H11	0.98
O7—H7	0.82	C13—C14	1.459 (5)
N1—C4	1.467 (3)	C13—H13A	0.97
N2—C9	1.371 (3)	C13—H13B	0.97
N2—C10	1.431 (3)	C14—H14A	0.96
N2—H2	0.86	C14—H14B	0.96
C1—C6	1.384 (3)	C14—H14C	0.96
C1—C2	1.390 (3)	C16—H16A	0.96
C1—C7	1.516 (3)	C16—H16B	0.96
C2—C3	1.381 (3)	C16—H16C	0.96
C2—H2A	0.93	C17—H17A	0.96
C3—C4	1.366 (3)	C17—H17B	0.96
C3—H3	0.93	C17—H17C	0.96
C4—C5	1.375 (4)		
			
C12—O4—C13	119.7 (2)	N2—C10—C11	107.09 (18)
C15—O6—C16	118.2 (2)	C18—C10—C11	111.8 (2)
C10—O7—H7	109.5	C12—C11—C10	115.27 (18)
O2—N1—O1'	122.1 (3)	C12—C11—C7	110.74 (18)
O1'—N1—C4	117.4 (2)	C10—C11—C7	108.39 (18)
O2—N1—C4	119.6 (2)	C12—C11—H11	107.4
O2—N1—O1	112.9 (7)	C10—C11—H11	107.4
O1—N1—C4	113.5 (6)	C7—C11—H11	107.4
C9—N2—C10	121.81 (19)	O3—C12—O4	125.6 (2)
C9—N2—H2	119.1	O3—C12—C11	124.7 (2)
C10—N2—H2	119.1	O4—C12—C11	109.7 (2)
C6—C1—C2	118.6 (2)	O4—C13—C14	109.4 (3)
C6—C1—C7	121.39 (19)	O4—C13—H13A	109.8
C2—C1—C7	119.82 (19)	C14—C13—H13A	109.8
C3—C2—C1	120.8 (2)	O4—C13—H13B	109.8
C3—C2—H2A	119.6	C14—C13—H13B	109.8
C1—C2—H2A	119.6	H13A—C13—H13B	108.2
C4—C3—C2	118.6 (2)	C13—C14—H14A	109.5
C4—C3—H3	120.7	C13—C14—H14B	109.5
C2—C3—H3	120.7	H14A—C14—H14B	109.5
C3—C4—C5	122.6 (2)	C13—C14—H14C	109.5
C3—C4—N1	118.8 (2)	H14A—C14—H14C	109.5
C5—C4—N1	118.6 (2)	H14B—C14—H14C	109.5
C4—C5—C6	118.0 (2)	O5—C15—O6	121.4 (2)
C4—C5—H5	121.0	O5—C15—C8	127.6 (2)
C6—C5—H5	121.0	O6—C15—C8	111.0 (2)
C5—C6—C1	121.3 (2)	O6—C16—H16A	109.5
C5—C6—H6	119.3	O6—C16—H16B	109.5
C1—C6—H6	119.3	H16A—C16—H16B	109.5
C1—C7—C8	114.77 (17)	O6—C16—H16C	109.5
C1—C7—C11	107.06 (17)	H16A—C16—H16C	109.5
C8—C7—C11	109.70 (17)	H16B—C16—H16C	109.5
C1—C7—H7A	108.4	C9—C17—H17A	109.5
C8—C7—H7A	108.4	C9—C17—H17B	109.5
C11—C7—H7A	108.4	H17A—C17—H17B	109.5
C9—C8—C15	120.6 (2)	C9—C17—H17C	109.5
C9—C8—C7	121.00 (19)	H17A—C17—H17C	109.5
C15—C8—C7	118.15 (19)	H17B—C17—H17C	109.5
C8—C9—N2	120.7 (2)	F1—C18—F3	107.1 (2)
C8—C9—C17	126.9 (2)	F1—C18—F2	107.5 (2)
N2—C9—C17	112.41 (19)	F3—C18—F2	106.9 (2)
O7—C10—N2	113.3 (2)	F1—C18—C10	112.3 (2)
O7—C10—C18	106.83 (19)	F3—C18—C10	111.6 (2)
N2—C10—C18	108.25 (19)	F2—C18—C10	111.2 (2)
O7—C10—C11	109.64 (18)		
			
C6—C1—C2—C3	1.0 (3)	O7—C10—C11—C12	−64.1 (3)
C7—C1—C2—C3	−174.7 (2)	N2—C10—C11—C12	172.51 (19)
C1—C2—C3—C4	−0.7 (4)	C18—C10—C11—C12	54.1 (3)
C2—C3—C4—C5	−0.2 (4)	O7—C10—C11—C7	60.6 (2)
C2—C3—C4—N1	−178.7 (2)	N2—C10—C11—C7	−62.8 (2)
O2—N1—C4—C3	−169.9 (3)	C18—C10—C11—C7	178.84 (19)
O1'—N1—C4—C3	20.9 (6)	C1—C7—C11—C12	−56.5 (2)
O1—N1—C4—C3	−32.6 (13)	C8—C7—C11—C12	178.39 (18)
O2—N1—C4—C5	11.5 (4)	C1—C7—C11—C10	176.15 (17)
O1'—N1—C4—C5	−157.7 (5)	C8—C7—C11—C10	51.0 (2)
O1—N1—C4—C5	148.8 (12)	C13—O4—C12—O3	5.3 (4)
C3—C4—C5—C6	0.7 (4)	C13—O4—C12—C11	−172.6 (2)
N1—C4—C5—C6	179.3 (2)	C10—C11—C12—O3	74.1 (3)
C4—C5—C6—C1	−0.4 (4)	C7—C11—C12—O3	−49.4 (3)
C2—C1—C6—C5	−0.4 (3)	C10—C11—C12—O4	−108.0 (2)
C7—C1—C6—C5	175.2 (2)	C7—C11—C12—O4	128.5 (2)
C6—C1—C7—C8	50.4 (3)	C12—O4—C13—C14	102.9 (3)
C2—C1—C7—C8	−134.0 (2)	C16—O6—C15—O5	3.2 (3)
C6—C1—C7—C11	−71.6 (3)	C16—O6—C15—C8	−178.7 (2)
C2—C1—C7—C11	104.0 (2)	C9—C8—C15—O5	16.0 (3)
C1—C7—C8—C9	−137.4 (2)	C7—C8—C15—O5	−169.7 (2)
C11—C7—C8—C9	−16.9 (3)	C9—C8—C15—O6	−161.96 (19)
C1—C7—C8—C15	48.4 (2)	C7—C8—C15—O6	12.3 (3)
C11—C7—C8—C15	168.92 (18)	O7—C10—C18—F1	171.4 (2)
C15—C8—C9—N2	166.91 (19)	N2—C10—C18—F1	−66.2 (3)
C7—C8—C9—N2	−7.1 (3)	C11—C10—C18—F1	51.5 (3)
C15—C8—C9—C17	−11.1 (3)	O7—C10—C18—F3	51.2 (3)
C7—C8—C9—C17	174.88 (19)	N2—C10—C18—F3	173.5 (2)
C10—N2—C9—C8	−6.4 (3)	C11—C10—C18—F3	−68.8 (3)
C10—N2—C9—C17	171.9 (2)	O7—C10—C18—F2	−68.1 (3)
C9—N2—C10—O7	−79.4 (3)	N2—C10—C18—F2	54.3 (3)
C9—N2—C10—C18	162.2 (2)	C11—C10—C18—F2	172.0 (2)
C9—N2—C10—C11	41.6 (3)		

### Hydrogen-bond geometry (Å, °)

**Table:**

*D*—H···*A*	*D*—H	H···*A*	*D*···*A*	*D*—H···*A*
O7—H7···O5^i^	0.82	1.98	2.783 (3)	166
N2—H2···O3^ii^	0.86	2.20	3.030 (3)	163
N2—H2···F2	0.86	2.42	2.735 (2)	102
C5—H5···O2^iii^	0.93	2.51	3.432 (4)	171

Symmetry codes: (i) −*x*+1/2, −*y*+1/2, −*z*+2; (ii) *x*, −*y*+1, *z*+1/2; (iii) −*x*, *y*, −*z*+3/2.
